# Epidural Labor Analgesia for a Patient with Neuromyelitis Optica: A Case Report and Review of the Literature

**DOI:** 10.1155/2018/2404756

**Published:** 2018-12-04

**Authors:** Alexandra Chang, Brian Chung, Rashmi Vandse

**Affiliations:** Anesthesia, Loma Linda University, Loma Linda, Ca, USA

## Abstract

Neuromyelitis optica (NMO) is a rare demyelinating disorder affecting the spinal cord and optic nerves. Like multiple sclerosis (MS), it predominantly affects women during childbearing years. The impact of neuraxial anesthesia on the course of NMO is uncertain. There are no large studies available to draw definitive conclusions regarding the safety of neuraxial anesthesia in this population. A review of the current literature suggests that neuraxial anesthesia is unlikely to exacerbate neurologic symptoms in pregnant patients with NMO. However, given the rarity of this disease entity among patients requesting epidural labor analgesia, we recommend taking a cautious approach.

## 1. Introduction

Neuromyelitis optica (NMO), or Devic's disease, is a rare autoinflammatory demyelinating disease of the optic nerves and spinal cord, characterized by optic neuritis and longitudinally extensive transverse myelitis [1, 2]. Lesions can develop at any spinal cord segment and may expand to encompass the brainstem, resulting in a range of symptoms including nausea, vomiting, intractable hiccups, loss of vision, weakness, numbness, bladder and/or bowel incontinence, respiratory failure, cognitive impairment, and autonomic dysregulation [2].

Given the inconsistent and unpredictable spinal cord involvement, offering epidural labor analgesia to a patient with NMO requires a judicious approach. This case report details the successful use of epidural labor analgesia in a patient with NMO who underwent induction of labor for intrauterine fetal demise (IUFD). We also present a review of the current literature pertaining to neuraxial anesthesia in patients with NMO.

## 2. Case Description

Our patient was a 24-year-old G2P0010 woman of Mexican descent who was admitted in August of 2017 for induction of labor for IUFD at 36 weeks of gestation. She was diagnosed with NMO in 2016 after initially presenting with symptoms concerning for area postrema syndrome, followed by magnetic resonance imaging (MRI) showing a spinal cord lesion from C2-T1 and a positive aquaporin-4 (AQP4) autoantibody. She was treated with a brief course of corticosteroids and azathioprine. Her past medical history was remarkable for recurrent syncope requiring placement of a permanent pacemaker in 2012 with a hospital course complicated by deep venous thrombosis of the common femoral vein and inferior vena cava (IVC), for which she received an IVC filter. She also had one prior therapeutic abortion at 9 weeks of gestation. Her home medications included subcutaneous heparin 10,000 units twice daily initiated one week prior to hospitalization for prophylaxis of venous thromboembolism, oxcarbazepine for muscle spasms, and prenatal vitamin. Labs on the day of admission showed hemoglobin of 9.9 grams/deciliter, platelet count of 223,000/microliter, fibrinogen of 491 milligrams/deciliter, international normalized ratio of 0.9, and partial thromboplastin time of 24 seconds. Transthoracic echocardiogram revealed normal ventricular and valvular function with no masses or cardiac source of emboli.

During preanesthesia evaluation, the patient reported occasional muscle spasms and positional double vision but denied weakness and neuropathy. Her airway, cardiovascular, and pulmonary examinations were normal. A neurological examination of cranial nerves, sensation, motor function, cerebellar function, and reflexes was unremarkable. The patient was educated on the risks and benefits of neuraxial anesthesia based on the available literature, including the remote possibility of exacerbation of neurological symptoms. She elected to first try intravenous opioids for pain control, including intravenous hydromorphone followed by patient-controlled analgesia with fentanyl. However, she had a protracted course of labor and eventually requested epidural analgesia on the third day. A coagulation profile was rechecked and found to be normal. A lumbar epidural catheter was placed at the L3-4 interspace. Following a negative test dose, 100 micrograms of fentanyl and 8 milliliters of 0.2% ropivacaine were loaded. Consistent with our institution's protocol, an epidural infusion was started using 0.15% ropivacaine and fentanyl 2.5 *μ*g/ml at 8 ml/hour with patient-controlled boluses of 8 ml every 20 minutes with a maximum dose of 2 boluses per hour. On that same afternoon, the patient delivered a demised neonate, and the cause of death was determined to be an umbilical cord accident. The patient was evaluated postpartum and reported no exacerbation in her pre-existing neurological symptoms. She was contacted via telephone at 5 months and 8 months after discharge, and she reported no change in her symptoms.

## 3. Discussion

Significant progress has been made over the past decade in elucidating the pathological, radiological, immunological, and clinical characteristics of NMO. Like MS, NMO predominantly affects women with a 9:1 ratio [2]. However, compared to MS, NMO is more frequent in non-Caucasians with a median age of onset in the late fourth decade [2]. The pathogenesis of NMO suggests the role of humoral immune mechanisms with the production of AQP4 antibodies and activation of the complement cascade [1–3]. The diagnoses of NMO and neuromyelitis optica spectrum disorder (NMOSD) include a combination of AQP4 antibody testing, MRI features, and core clinical characteristics such as optic neuritis, acute myelitis, area postrema syndrome, acute brainstem syndrome, narcolepsy, and cerebral syndrome [1, 2]. Patients typically present with an acute episode of optic neuritis or transverse myelitis, and the majority of patients develop a relapsing course, often with severe residual deficits rapidly leading to disability [1, 2]. The overall 10-year mortality rate is 20–25% and is typically due to neurogenic respiratory failure [1]. Acute episodes are generally treated with intravenous glucocorticoids and plasma exchange followed by long-term immunosuppression [2].

Pregnancy has a negative effect on the progression of NMO, possibly due to increased humoral immunity and TH2 cytokines [4, 5]. Four review studies found an increased rate of relapse of NMO during the first three months postpartum [4, 6–8]. In a review of 139 Japanese women with NMOSD, the study authors noted an increased annualized relapse rate (ARR) during the first three months postpartum compared to pre-pregnancy; however, the ARR was not reduced during pregnancy [8]. In another review of 190 women with NMOSD involving 54 pregnancies, the ARR increased significantly during the first six months after delivery with 77% of pregnancies resulting in postpartum relapses [9]. It is believed that insufficient immunosuppression may increase the risk of exacerbation during pregnancy [8–10]. Since the risk of relapse after delivery appears to be high, patients with NMO are recommended to undergo treatment immediately after delivery [10].

Literature encompassing the anesthetic management of pregnant patients with NMO is limited and yields conflicting results. Published case reports and case series discussing the effects of neuraxial anesthesia include fewer than 50 pregnant patients with NMO or NMOSD [4–6]. Many published cases do not detail the type and concentration of medications used for neuraxial anesthesia. In a review of 13 patients with NMO, no correlation was found between epidural labor analgesia and NMO activity [4]. In another review of 17 patients with NMO, 5 patients underwent uneventful spinal anesthesia [6]. Another case report describes the use of spinal anesthesia for cesarean delivery during an acute exacerbation of NMO [5]. Interestingly, the patient's neurologic symptoms resolved after the delivery.

In contrast, some case studies have suggested the possibility of exacerbation of NMO by the neuraxial anesthesia [11–13]. The susceptibility of demyelinated nerves to local anesthetic toxicity was hypothesized to have caused severe tetraparesis in a pregnant NMO patient, who underwent cesarean delivery under spinal anesthesia [11]. However, there is no clinical data to support this assumption. Another patient developed NMO after spinal anesthesia for orthopedic surgery [12]. Another case report describes a patient who received epidural anesthesia for a cesarean delivery and developed NMO two months later [13]. It has been suggested that neuraxial anesthesia may simply unmask the latent NMO instead of causing it.

Though MS is a distinct entity from NMO, the current literature has similarly shown no relationship between exacerbations of MS and neuraxial anesthesia. Two prospective studies evaluating the safety of epidural anesthesia in obstetric patients failed to show any negative outcomes [14]. A systematic review of 231 patients with MS also found no evidence that neuraxial anesthesia negatively affects the course of MS [14]. There are only case reports about spinal anesthesia and MS, but they also do not show a clear negative relationship. A retrospective review of 139 neuraxial procedures performed on patients with a broad spectrum of central nervous system disorders, including multiple sclerosis, found that the apprehensions about neuraxial anesthesia in this patient population may be overstated [15].

Our case was further complicated by the patient's IUFD, which heightened our team's sensitivity to her psychosocial state. It was important to our team that we help alleviate her physical pain in order to minimize any additional psychosocial burden. Ultimately, our hesitancy to perform the epidural procedure due to NMO may have influenced the content of our risk-benefit discussion, resulting in the patient's initial decision to control her pain with intravenous opioids.

This case report illustrates the safe and effective use of epidural labor analgesia in a patient with NMO, lending support to the current opinion in literature that it does not cause nor aggravate NMO, though a lack of randomized controlled trials on this subject limit our ability to make definitive conclusions. We believe that the rarity of NMO among patients requesting epidural analgesia necessitates the following cautious approach: (1) in-depth discussion about the increased risk of NMO relapse during pregnancy and in the postpartum period, as well as further discussion about the natural course of NMO whereby most patients develop a relapsing pattern; (2) detailed documentation of the patient's baseline neurologic exam; and (3) postpartum follow-up with the patient. The decision to proceed with epidural analgesia should be made on an individualized basis after ensuring the patient's understanding of the procedure's risks and benefits. Due to the concern, though perhaps unfounded, that demyelinated or abnormal spinal cord segments may be more sensitive to local anesthetic toxicity, we also recommend avoiding spinal anesthesia when possible and using a dilute concentration of local anesthetic with narcotics for epidural labor analgesia.
